# Peripheral Odontogenic Fibroma: A Report of a Rare Case and Review of Literature

**DOI:** 10.7759/cureus.58154

**Published:** 2024-04-12

**Authors:** Alka Hande, Padmashri P Kalmegh, Archana M Sonone, Swati K Patil, Preethi N Sharma, Aayushi Pakhale

**Affiliations:** 1 Oral Pathology and Microbiology, Sharad Pawar Dental College and Hospital, Datta Meghe Institute of Higher Education and Research, Wardha, IND; 2 Oral and Maxillofacial Pathology, Sharad Pawar Dental College and Hospital, Datta Meghe Institute of Higher Education and Research, Wardha, IND

**Keywords:** recurrence, benign tumor, odontogenic tumors, odontogenic islands, peripheral odontogenic fibroma

## Abstract

Peripheral odontogenic fibroma (POF) is described as a relatively rare, benign, extraosseous odontogenic tumor derived from odontogenic ectomesenchyme. It is characterized by a mature fibrous stroma with embedded inactive resting islands of odontogenic epithelium. In the category of peripheral/extraosseous neoplasms, odontogenic fibroma (OF) is one of the most prevalent tumors. The radiographic examination shows minimum bone loss in the alveolar crest area. It poses a diagnostic challenge for clinicians and pathologists because its clinical and radiological aspects are similar to other peripheral odontogenic as well as non-odontogenic tumors, and the differential diagnosis is predicated on histological assessment. Histopathological examination is the key to a final confirmed diagnosis. This article presents a case report of a 53-year-old male who reported a painless, pale pink mass in the maxillary anterior region. We emphasize the clinicopathological, radiographical, and histopathological aspects of the rare entity of POF.

## Introduction

Odontogenic tumors typically arise as intraosseous pathology. However, occasionally they may appear as extraosseous lesions commonly seen on the gingiva [1]. Odontogenic fibroma (OF) is a relatively uncommon neoplasm, derived from odontogenic ectomesenchyme. It has been found that there are two topographical variants: central (intraosseous) and peripheral (extraosseous) [2]. Peripheral odontogenic fibroma (POF) is a relatively rare tumor characterized by a mature fibrous stroma with embedded inactive/resting islands of odontogenic epithelium [3]. POF comprises approximately 1.2% to 4.7% of all odontogenic tumors [4]. It most commonly occurs at a range between five months to 84 years, with a peak in the third decade. This is an uncommon, benign, encapsulated ectomesenchymal tumor that is most commonly seen in adult females, having a prevalence in the mandible [5]. Clinically lesion appears as an asymptomatic entity but sometimes tooth displacement may be the primary complaint. However, the prevalence of involvement of the labial or buccal aspect of the maxilla as well as the mandible has also been reported [6]. The histopathological spectrum of the tumor comprises interlacing collagen fiber bundles in intercellular connective tissue stroma. Odontogenic epithelium in the form of long strands or nests throughout the lesion is the prominent component. Varying degrees of calcification composed of cementum-like material or dentinoid are present in the same area [5]. Radiolographical findings show the superficial erosion of the alveolar bone and tooth displacements infrequently. However, it does not involve the underlying bone [7]. Conservative local surgical excision is the current therapeutic choice for POF followed by long-term follow-up [4].

## Case presentation

**Figure 1:**
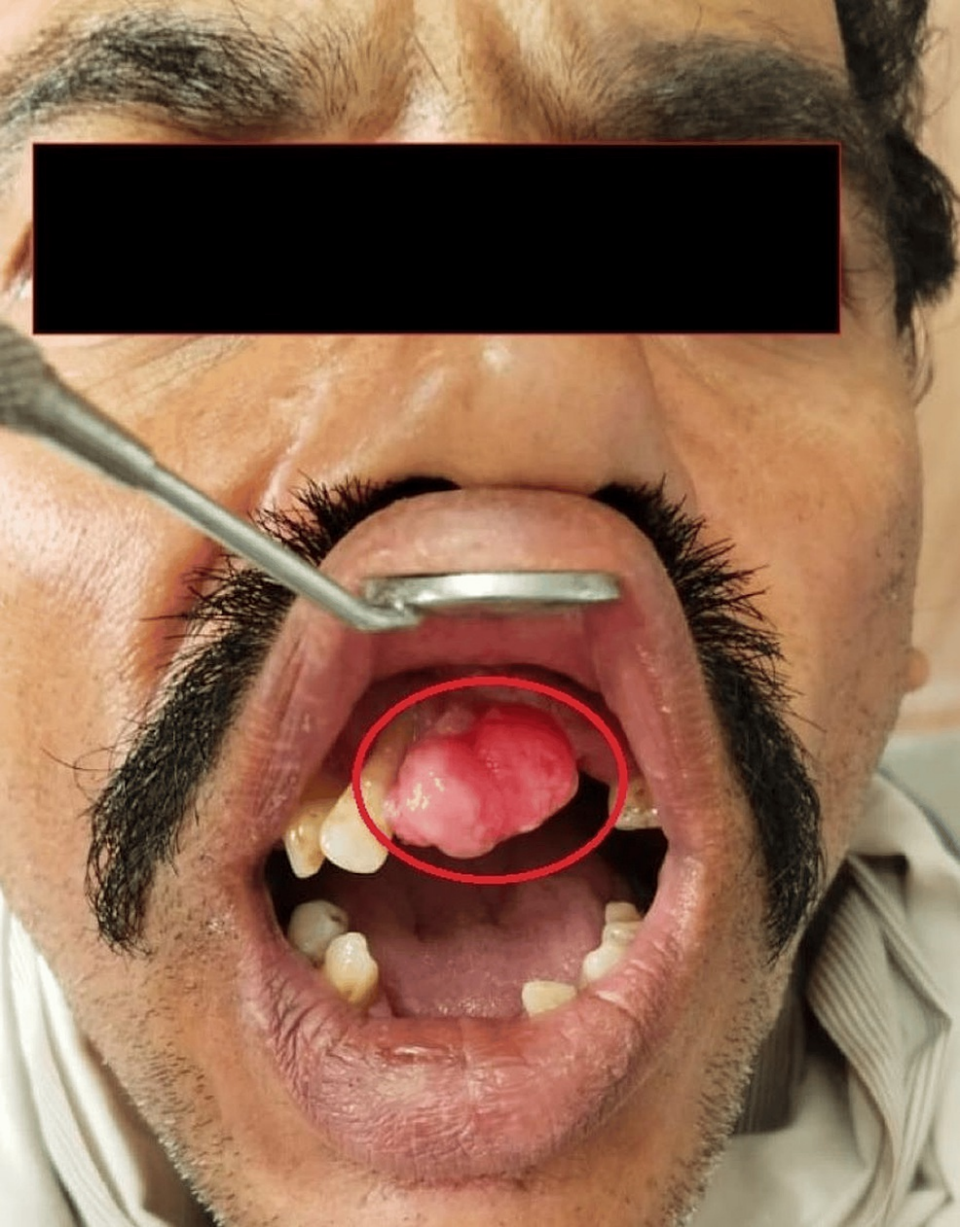
Intraoral lesion in the maxillary anterior region

A 53-year-old male patient presented to our institute with a complaint of a growth in the anterior region of the upper jaw for eight months The history of the present illness reveals the painless growth of a small peanut size, which gradually grew to the present size. On extraoral examination, facial asymmetry was not evident. Intraoral examination revealed a smooth, pale pink, non-inflammatory, painless, firm, sessile, well-defined mass measuring 2.3 x 2 cm in size, extending from 12 to 22 region (Figure 1). 

**Figure 2:**
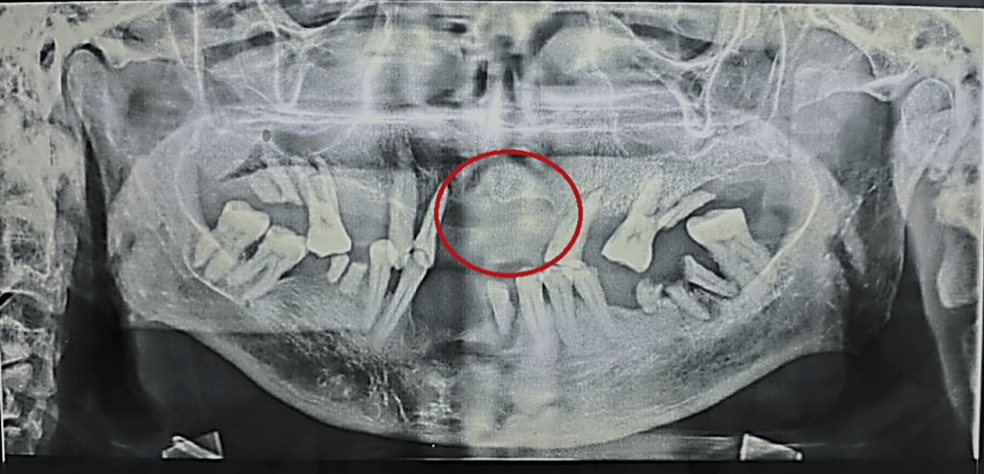
Orthopantomogram showing bone loss with foci of calcifications

The teeth adjacent to the growth showed the presence of plaque and calculus and involvement of periodontal tissues, however, mobility was not observed. There were missing 11, 21, 22, 23, 24, and 26. No relevant past history was reported by the patient. Orthopantomagram showed bone loss/erosion with dispersed radiopaque foci (Figure 2).

**Figure 3:**
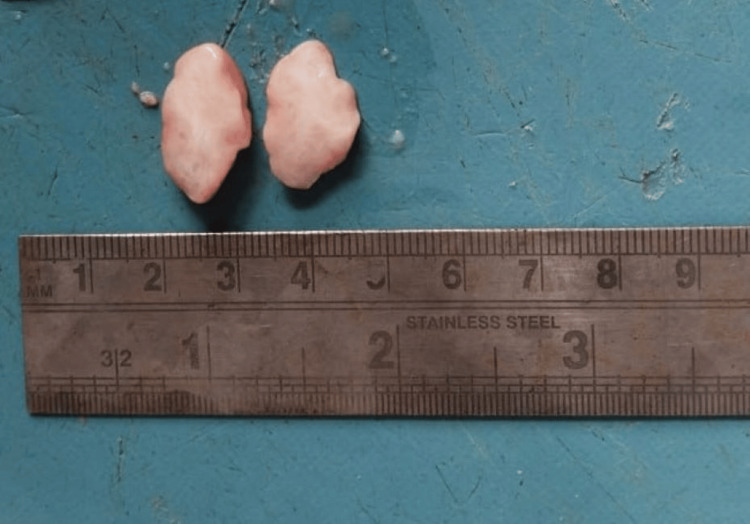
Macroscopic specimen showing multiple, oval, pinkish, soft tissue specimens measuring 2 x 1.8 x 1 cm in size

Displacement and resorption of adjacent teeth were observed. A biopsy was carried out under local anesthesia and further processed for histological diagnosis (Figure 3).

**Figure 4:**
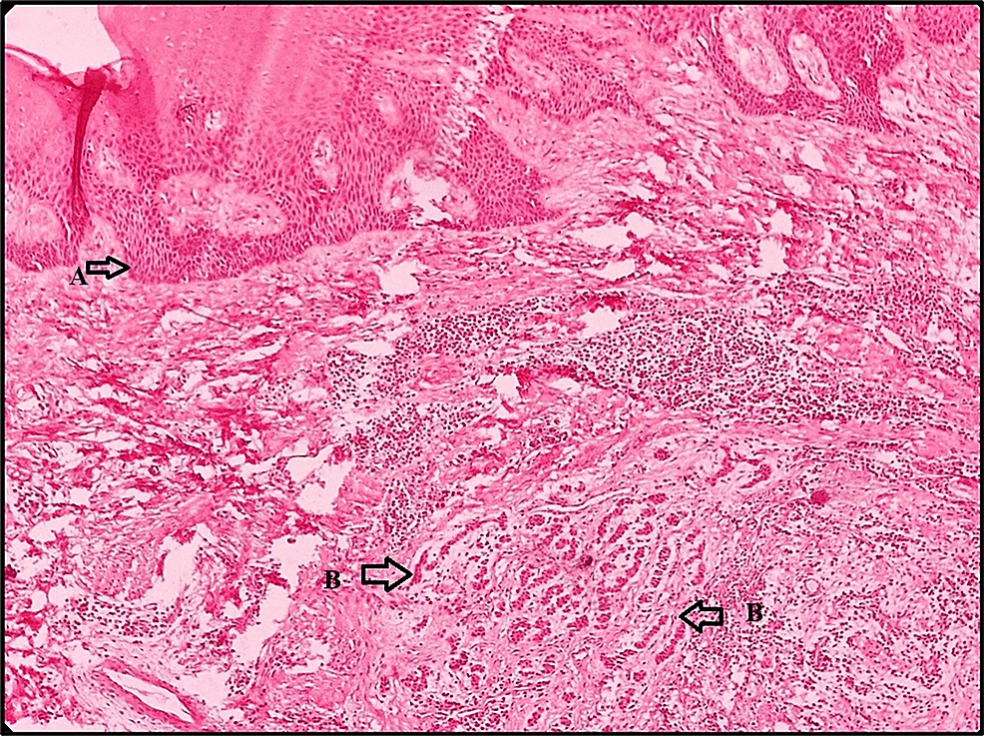
Hematoxylin and eosin stained section showing overlying proliferative epithelium (A) and underlying fibrocellular connective tissue stroma (B)

On microscopic examination, the hematoxylin and eosin (H&E) stained section revealed the presence of overlying proliferative epithelium of stratified squamous type and underlying fibrocellular connective tissue stroma (Figure 4).

**Figure 5:**
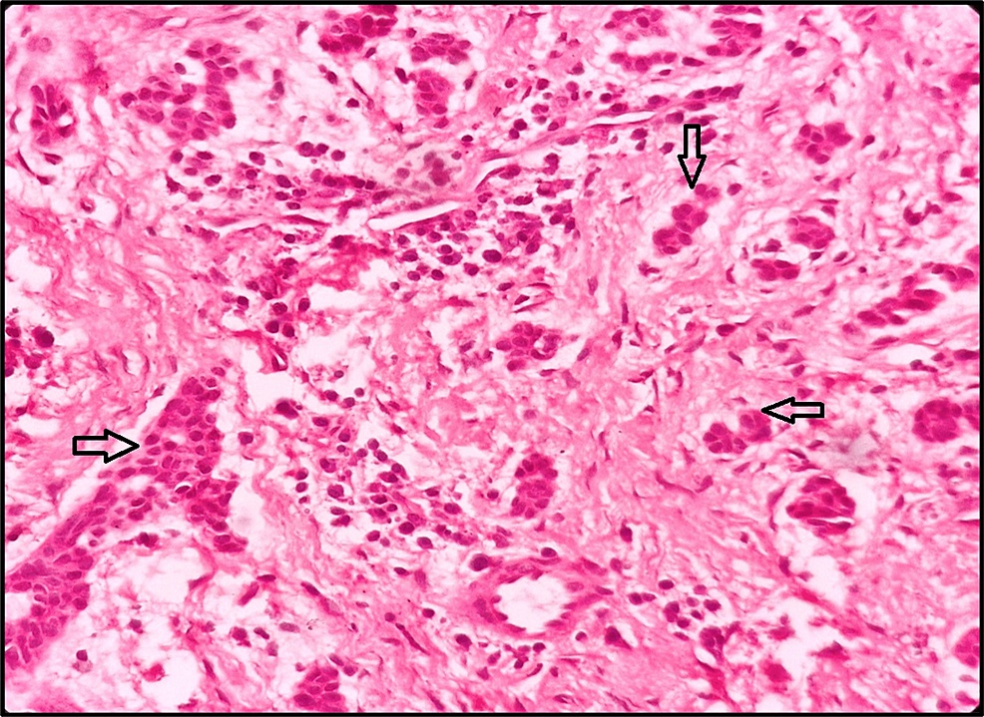
Hematoxylin and eosin stained section showing inactive odontogenic islands (black arrows) in the fibrocellular connective tissue stroma

There was the presence of multiple and variable-sized inactive odontogenic islands comprising cuboidal cells with centrally placed, hyperchromatic nuclei in the fibrocellular connective tissue stroma (Figure 5).

**Figure 6:**
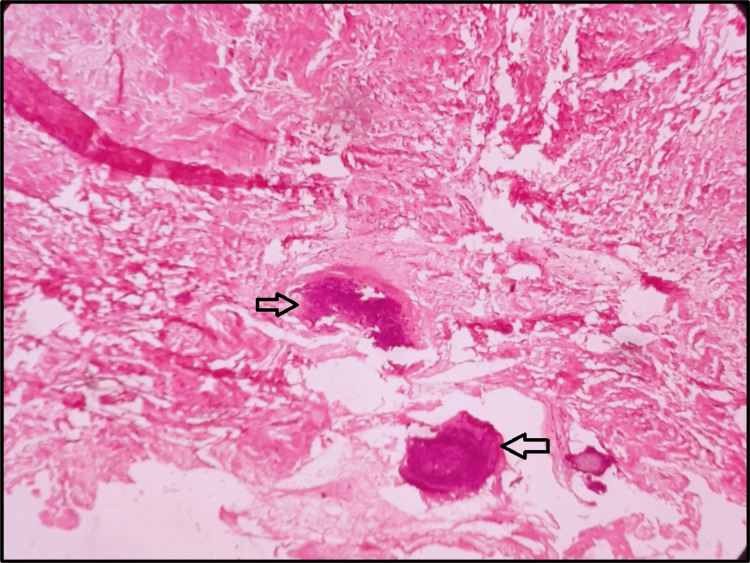
Hematoxylin and eosin stained section showing calcifications (black arrows) in the fibrocellular connective tissue stroma

Variable calcified bodies resembling dysplastic dentin or cementum-like tissues were also evident (Figure 6).

All these clinical, histopathological, and radiographical pathognomonic features were suggestive of POF. The treatment plan of the pathology comprised surgical excision of the lesion followed by curettage and post-surgical follow-up for three months, which showed no clinical evidence of relapse.

## Discussion

OF is a benign, uncommon neoplasm comprising resting inactive islands of odontogenic epithelium in a fibrocellular connective tissue stroma with the presence of calcifications [7]. According to Gardner, there are two categories of OFs: central and peripheral [5]. In 1971, the World Health Organization (WHO) classified POF as an extraosseous counterpart of central OF, which is a fibroblastic tumor with dentin and/or cementum-like tissue formation. POF and ossifying fibroma terms were used interchangeably until 1982 when Gardner differentiated them as a separate entity. Subsequently, in 1992, the WHO classified it as an odontogenic tumor, which might comprise either odontogenic epithelium or odontogenic ectomesenchyme [8]. Though the histogenesis of the POF has yet to be determined, it is widely acknowledged that this lesion is an odontogenic tumor of ectomesenchymal origin. The periodontal ligament, ectomesenchyme, dental lamina rests, and surface epithelium have all been proposed as potential sources of the lesion. As per a literature search, the POF may have originated from remnants of the dental lamina [9].

Farman claims that the epithelial ectomesenchymal interaction may cause the remnants of the dental lamina and basal layer of the gingiva to undergo secondary growth [10]. Histopathologically POF is characterized by dispersed islands of odontogenic epithelium in the fibrocellular stroma. Depending on the amount of epithelium present, a subtype can be classified as either epithelium-rich or epithelium-poor. Moreover, hard tissue formation resembling 28.3% of bone-like tissue, and 15.2% of dentin or cementum-like tissue is typically seen [5]. The mandible and anterior maxilla are recognized to be the most common locations for the occurrence of POF in adults, despite a few studies suggesting the prevalence in the mandibular canine to premolar region [5]. The ratio of females to males may range from 1.1:1 to 7.5:1, and the majority of literature indicates that these tumors mostly affect females [4]. Clinically, POF presents as a slow-growing, asymptomatic, non-ulcerated nodule or round, irregular mass that grows slowly and typically ranges in diameter from 0.5 to 3.5 cm [7].

POF rarely affects the underlying bone, hence radiographic alterations are uncommon. As a result, Ritwik and Brannon documented radiographic findings in only 12 out of the 151 patients of POF. It does not involve underlying bone and may show areas of calcification. The most prevalent radiographic findings of POF are zones of superficial bone erosion along with horizontal bone loss. Very infrequently it may induce resorption of alveolar bone and displacement of adjacent teeth [11]. Since the clinical appearance is similar to other gingival overgrowths, gingival epithelial hamartoma, focal gingival fibrous hyperplasia, pyogenic granuloma, peripheral giant cell granuloma, peripheral ossifying fibroma, peripheral calcifying epithelial odontogenic tumor, and peripheral ameloblastoma should be considered as differential diagnosis [6]. We need to differentiate these lesions histologically. Previously, it was referred to as odontogenic gingival epithelial hamartoma because the authors emphasized the epithelial component higher than the fibroblastic component [1]. Compared to peripheral ameloblastoma, the odontogenic epithelial islands of POF are inactive, resting, and smaller. Odontogenic epithelial rests are commonly observed in POF but not in peripheral ossifying fibroma. Peripheral giant cell granuloma contains more multinucleated giant cells than POF. In contrast to POF, pyrogenic granuloma exhibits vascular proliferations resembling granulation tissue [12].

Surgical excision is being considered as the main course of therapy. However, as per the literature review, post-therapy recurrence of POF should be taken into consideration [7]. Several studies have found a 3.3% to 5.5% rate of recurrence However, certain retrospective researches reveal a high risk of local recurrence, in 17.6% to 50% of cases. The relapse of POF is mostly prevalent for the initial first two years post-therapy [4]. The recurrence is linked to two histopathological parameters: (i) budding of basal cells and (ii) calcifications, rather than odontogenic epithelial rest.

## Conclusions

POF is a relatively rare, slow-growing, asymptomatic, benign odontogenic tumor that is frequently misinterpreted as an inflammatory condition. Because of its rarity, the lesion is not frequently mentioned in the literature. Despite having many characteristics in common with various other gingival lesions, some aspects can assist physicians and pathologists in making an accurate diagnosis. As a result, a histological diagnosis is required to differentiate it from others. It exhibits potential for expansion and recurrence. Although it is thought to recur following excision, the exact recurrence rate is unknown due to a lack of evidence in the literature. As a result, it is essential to maintain regular post-operative follow-up.
